# Cytotoxicity of white birch bud extracts: Perspectives for therapy of tumours

**DOI:** 10.1371/journal.pone.0201949

**Published:** 2018-08-14

**Authors:** Valery Isidorov, Łukasz Szoka, Jolanta Nazaruk

**Affiliations:** 1 Forest Faculty, Bialystok University of Technology, Hajnówka, Poland; 2 Department of Medicinal Chemistry, Medical University of Bialystok, Bialystok, Poland; 3 Department of Pharmacognosy, Medical University of Bialystok, Bialystok, Poland; College of Agricultural Sciences, UNITED STATES

## Abstract

Birch buds (*Gemmae Betulae*) are widely used in Russian and Chinese traditional medicine mainly as a diuretic and diaphoretic agent but also as an antiseptic, anti-inflammatory and analgesic. Despite the long history of therapeutic use of birch buds in folk medicine, the existing information on their chemical composition and pharmacological effects is insufficient. This circumstance warrants further study of the chemistry and pharmacology of birch buds. The present study was designed to investigate (a) the chemical composition of buds from two species of white birch and (b) the *in vitro* cytotoxic effect of extracts from these sources on selected tumour cells. Extracts from *Betula pubescens* Ehrh. and *Betula pendula* Roth. buds were obtained using three different methods: carbon dioxide supercritical fluid extraction (SFE), washing of exudate covering whole buds, and extraction of milled buds with diethyl ether. The chemical composition of extracts was investigated by GC-MS. Cytotoxicity was determined by MTT assay, and cell proliferation was determined by [^3^H]thymidine uptake in cancer cells and normal skin fibroblasts. The GC-MS investigation identified a total of 150 substances of different classes. The chemical composition of *B*. *pubescens* and *B*. *pendula* buds differed, with bud extracts from the former containing a relatively high quantity of sesquiterpenoids and flavonoids, while the main components of extracts from the latter were triterpenoids. The results of the biological assay indicated that birch bud extracts demonstrated time- and concentration-dependent and differential cytotoxicity. The highest cytotoxic activity demonstrated bud exudates and SFE extracts obtained from both *Betula* species. The rich chemical composition of birch buds suggests the possibility of a wider spectrum of biological activity than previously thought. Birch bud extracts could be a promising source of compounds with cytotoxic activity against various cancers.

## Introduction

The birch (*Betula* L.) is one of the main arborous plants in the forests of boreal and temperate zones as well as the mountain regions of the Northern Hemisphere [1]. It is a medicinal plant that has been used in traditional medicine since ancient times. Its traditional use is well documented in the ethnobotanical literature [2–11]. Leaves, buds, tar and essential oils are used to treat a wide spectrum of diseases, including inflammation, infections, urinary tract disorders, skin and hair problems [12–14]. In Polish folk medicine, ethanolic maceration of fresh buds from *B*. *pendula* was used on bleeding wounds [15]. Buds collected in the winter were taken instead of leaves as a diuretic remedy [5]. In Russian folk medicine, ethanolic macerations were used internally to treat stomach disorders and fever and externally to treat rheumatism [16].

In modern Russia (as well as in the former USSR), an assemblage of birch buds, *Gemmae Betulae*, is a standardised medical preparation [17]. The scientifically proven health benefits of bud extracts are primarily due to their diuretic effect [9,11] and antimicrobial and antioxidant properties [10,18–20]. Only anecdotal publications have been devoted to the anticancer activity of birch buds [21–23]. Despite the wide usage of birch buds in folk medicine and growing interest of conventional medicine in this herbal material, the current information on its chemical composition is insufficient for medical purposes.

One of the main goals of this investigation was to fill this gap by determining the chemical composition of birch buds. The *Gemmae Betulae* preparation is described as a mixture of *Betula pendula* Roth. and *Betula pubescens* Ehrh. (*Betulaceae*) buds, but the proportions are not regulated. However, substantial differences in the chemical compositions of the resins covering the buds of these closely related species were recently demonstrated [24]. Moreover, it is well known that the chemical composition of any plant-derived preparation (and, as a consequence, its biological activity), in many respects, depends on the procedure used for extraction. For this reason, we used three different extraction procedures for buds from each of the white birch species.

The second main goal was to evaluate the anticancer potential of these extracts to examine the relationship between composition and antitumour activity, and to identify extracts worthy of further investigation.

## Materials and methods

### Reagents and chemicals

Dulbecco's modified Eagle's medium (DMEM), Minimum Essential Medium (MEM), Roswell Park Memorial Institute 1640 Medium (RPMI 1640), fetal bovine serum (FBS), phosphate-buffered saline (PBS), sodium pyruvate, trypsin, penicillin and streptomycin were obtained from Gibco (Thermo Fisher Scientific, Waltham, MA, USA). [^3^H]thymidine was purchased from Hartmann Analytic (Braunschweig, Germany). Sodium dodecyl sulfate (SDS) was obtained from Bio-Rad Laboratories (Hercules, CA, USA). Dimethyl sulfoxide (DMSO), 3-(4,5-dimethylthiazole-2-yl)-2,5-diphenyltetrazolium bromide (MTT), glycine, sodium chloride, sodium hydroxide and cisplatin were purchased from Sigma-Aldrich (Saint Louis, MO, USA).

### Plant material

Buds of downy birch (*Betula pubescens* Ehrh.) and silver birch (*Betula pendula* Roth.) were gathered in August–September 2015 from trees growing in the Biebrza National Park in northeastern Poland (53° 32' N, 22° 43' E). Voucher specimens (no BP-17034 and BO-17035) have been deposited with the herbarium of the Department of Pharmacognosy, Medical University of Bialystok (Poland). A previously described method was used to identify the birch species [25]. Plant material was kept at -18°C before use.

### Sample preparation and chemical analysis

Carbon dioxide supercritical fluid extraction (SFE) of buds was performed in October 2015 in the High Pressure Technique Laboratory of the Supercritical Extraction Department in the New Chemical Syntheses Institute (Puławy, Poland). Experimental parameters were as follows: extraction pressure 300 bar, temperature 40°C; yield of the product was about 7.2%. Extracts were light-yellow and slightly viscous with the characteristic birch fragrance.

Exudates covering the buds from each of the birch species were extracted by intensive rinsing of the bud samples (15–20 g) for 60 s in diethyl ether (50 mL). The extracts were filtered through the paper filter, and the solvent was evaporated to dryness.

The washed buds were milled and immediately transferred into a retort of 250 mL in volume and extracted under constant stirring, using three volumes of 50 mL of diethyl ether. The duration of each extraction cycle at room temperature was 30 min. The combined diethyl ether extracts were filtered through a paper filter and the solvent was removed on a rotor evaporator.

About 5 mg of the residue of exudate and extract left on the walls (as well as 5 mg of SFE products) was put into a vial of 2 mL in volume. After dissolving in 220 μL of pyridine, 80 μL of BSTFA was added to the vial. The reaction mixture was sealed and heated for 0.5 h at 60 ^o^C to obtain trimethylsilyl (TMS) derivatives. The whole procedure was performed in triplicate.

The resulting solutions were separated and analysed by GC–MS on a HP 7890A gas chromatograph with the 5975C VL MSD Triple-Axis Detector (Agilent Technologies, USA). The apparatus was fitted with an HP-5MS capillary column (30 m × 0.25 mm i. d., 0.25 μm film thickness), with electronic pressure control and split/splitless injector. The latter worked at 250°C in the split (1:50) mode. The helium flow rate through the column was 1 mL/min in constant flow mode. Injection of 1 μL of the sample was performed with the aid of a G4513a autosampler. The injector (250 ^o^C) worked in split mode (1:50). The initial column temperature was 50 ^o^C rising to 310 ^o^C at 5 ^o^C/min. The MSD detector acquisition parameters were as follows: the transfer line temperature was 280 ^o^C, the MS source temperature 230 ^o^C and the MS quad temperature 150 ^o^C. The electron impact mass spectra were obtained at 70 eV of ionization energy. Detection was performed in the full scan mode from 41 to 650 a.m.u. After integration, the fraction of separated components in the total ion current (TIC) was calculated.

To identify the components, both mass spectral data and the calculated retention indices were used. Mass spectrometric identification was carried out with an automatic system of GC–MS data processing supplied by NIST and home-made mass spectra libraries. The latter contains more than 1800 spectra of TMS derivatives prepared from authentic preparations of flavonoids and other phenolics, as well as terpenoids, aliphatic acids, alcohols and carbohydrates.

The hexane solution of C_10_–C_40_ *n*-alkanes was separated under the above conditions. The linear temperature programmed retention indices (*I*^T^) of the registered components were calculated from the results of the separation of this solution and silanized bud extracts and were compared with the NIST collection [26] as well as with the authors' previously published data [24,27–29]. The identification was considered reliable if the results of the computerized search of the mass spectra library were confirmed by the experimental *I*^T^ values, i.e. if their deviation from the averaged published values did not exceed ±10 u.i. (for more information see Supplementary Information, S1 Text).

### Cell culture

Human breast adenocarcinoma MCF-7 and MDA-MB-231 cells, human colorectal adenocarcinoma DLD-1 cell line, human melanoma C32 cells, human gastric adenocarcinoma AGS cells, human glioblastoma cell lines LN-18 and LN-229 and human skin fibroblasts CCD-25Sk were obtained from ATCC (Manassas, VA, USA). The human endometrial adenocarcinoma cell line (Ishikawa), human cervix adenocarcinoma HeLa and human hepatocellular carcinoma HepG2 cells were purchased from Sigma-Aldrich. The cells were cultured in DMEM (except for the DLD-1 cells for RPMI 1640 and for the HeLa and HepG2 cells for MEM) supplemented with 10% FBS, 100 units/mL penicillin and 100 μg/mL streptomycin in a humidified 5% CO_2_ atmosphere at 37°C.

### Cytotoxicity assay

The viability of cells was determined by MTT assay [30]. Cells were detached with 0.25% trypsin and seeded at 1 × 10^4^ cells per well in 96-well plates. After reaching confluence, the tested extracts and anticancer drugs used as positive controls were added. Extracts were dissolved in DMSO, diluted with fresh medium and placed into 96-well plates at a volume of 200 μL per well. The final DMSO concentration did not exceed 0.1%. Control cells were cultured in medium containing 0.1% DMSO. Cisplatin was dissolved in medium. After 48 h, 100 μL of a 0.4% MTT solution in PBS was added to each well for 4 h. The medium was removed and the formazan crystals were dissolved in 200 μL of DMSO and 25 μL of Sorensen's glycine buffer for 10 minutes on a plate shaker. The optical density was measured in a microplate reader (Biochrom, Cambourne, United Kingdom) at 570 nm.

### [^3^H]thymidine incorporation assay

[^3^H]thymidine incorporation was used as a measurement of cell proliferation. Cells were seeded at 1× 10^4^ cells per well in 24-well tissue culture plates with 1 mL of growth medium. After 24 h, the cells were incubated with various concentrations of extracts or cisplatin and 0.5 μCi of [^3^H]thymidine for 24 h. Then, the medium was removed and cells were washed three times with ice-cold PBS and lysed in 1 mL of 0.1 M sodium hydroxide containing 1% SDS. The cell lysates were transferred to scintillation vials and 3 mL of the scintillation fluid (Perkin Elmer, Waltham, USA) was added. The amount of [^3^H]thymidine incorporated into the DNA was determined in a scintillation counter (Perkin Elmer).

### Statistical analysis

The results are presented as means ± SEM of at least two independent experiments. Differences between means for extracts-treated groups and vehicle-treated groups were analyzed using one-way ANOVA followed by Tukey’s test. P < 0.05 was considered statistically significant. IC_50_ values were calculated using nonlinear regression analysis using GraphPad Prism version 7.04 (GraphPad Software, La Jolla, CA, USA).

## Results

### Composition of extracts from buds of silver birch and downy birch

In this work, we used different extraction procedures: carbon dioxide supercritical fluid extraction, washing of bud resin exudate with diethyl ether, and extraction of milled buds by diethyl ether. It was hypothesised that the chemical composition of these extracts would differ, and that this difference may be reflected in their biological activity.

In line with expectations, the extraction procedures had a substantial influence on the composition of extracts at both the quantitative and qualitative level. In total, the GC analysis recorded 150 compounds with a relative content not less than 0.01% of the total ion current. Extracts from downy birch and silver birch buds contained 118 and 78 substances, respectively. Moreover, different extracts differed in the number of constituents: 87, 90, and 102 compounds in SFE, exudate and milled downy birch bud extracts, respectively. Silver birch buds contained 60, 74, and 64 compounds in the same extracts, respectively. Table 1 shows the group composition of extracts and the relative content of the main representatives of these groups. The relative composition of individual compounds and their analytical parameters (*I*^T^ values, *m/z* of target ions, and molecular ions, M^+^) are presented as Supplementary Information (S1 Fig, S1 Table).

**Table 1:** Group composition (% of TIC) of different extracts from white birches buds.

Compounds	*B*. *pubescens*			*B*. *pendula*		
	SFE	exudate	extract	SFE	exudate	extract
**Sesquiterpenoids**	**56.14**	**38.79**	**29.81**	**4.63**	**5.06**	**4.34**
including:						
β-caryophyllene	0.36	0.11	0.13	0.09	0.17	0.40
birkenal	0.43	1.01	0.42	-^a^	-	-
6-hydroxy-β-caryophyllene	12.54	5.93	5.80	1.48	2.85	238
6-hydroxy-β-caryophyllene acetate	2.28	1.07	0.79	0.31	trace	-
14-hydroxy-β-caryophyllene	3.49	1.45	1.57	045	0.77	062
14-hydroxy-β-isocaryophyllene	2.12	078	0.75	-	0.39	0.71
14-hydroxy-β-caryophyllene acetate	10.72	5.47	3.97	1.60	trace	-
**Triterpenoids**	**2.43**	**1.33**	**2.02**	**67.53**	**78.97**	**79.55**
including:						
dammaradien-3-one	-	-	-	5.01	5.00	4.08
dipterocarpol	0.12	-	0.07	7.06	8.90	8.33
lupen-20(29)-en-28-al^b^	-	-	-	16.01	28.35	35.29
betulinic acid	-	-	0.06	-	0.02	1.74
**Flavonoids**	**23.58**	**48.05**	**56.93**	**1.31**	**4.99**	**4.13**
including:						
apigenin	-	0.68	1.08	-	-	-
sakuranetin	6.14	14.60	12.50	0.13	0.33	0.63
kumatakenin	1.96	4.5	3.25	-	-	-
3'-methoxyapigenin	0.54	4.69	6.45	-	0.34	0.46
rhamnocitrin	0.36	3.19	4.78	-	0.10	0.12
pectolinaringenin	0.57	4.60	7.94	-	0.45	0.19
cirsimaritin	-	1.44	1.75	0.46	0.99	0.86
catechin	-	-	trace	0.29	0.35	0.28
**Phenylpropenoids**	**4.77**	**4.85**	**7.48**	**0.87**	**0.16**	**0.83**
including:						
6-hydroxycaryophyllene *p*-coumarate	1.70	2.29	3.54	-	-	-
14-hydroxycaryophyllene *p*-coumarate	1.01	0.94	2.25	-	-	-
6-hydroxycaryophyllene ferulate	0.16	0.18	0.16	-	-	-
14-hydroxycaryophyllene caffeate	0.13	0.20	0.22	-	-	-
*n*-docosyl *p*-coumarate	0.70	0.50	0.47	0.87	0.16	0.83
**Aromatics**	**-**	**-**	**0.57**	**0.37**	**0.38**	**0.24**
**Aliphatic C**_**16**_**-C**_**28**_ **acids and esters**	**6.27**	**0.73**	**1.13**	**11.28**	**8.21**	**8.60**
**Aliphatic C**_**20**_**-C**_**28**_ **alcohols**	**1.15**	**0.38**	**0.53**	**0.88**	**1.60**	**0.76**
**Alkanes**	**3.95**	**3.08**	**1.46**	**5.80**	**0.76**	**2.58**

^a^—not detected

^b^ identified tentatively based on the MS fragmentation patterns.

As can be seen from the data in Table 1, sesquiterpenoids were the main group of compounds in downy birch, while in silver birch buds, triterpenoids prevailed. The second main group found in downy birch extracts was flavonoids (24‒57% of TIC), but these were found at substantially lower levels in silver birch buds (1.3‒4.9% of TIC). Flavonoids in birch buds were presumably methoxylated flavones and 3-hydroxyflavones. Catechin, a flavan-3-ol, was detected in noticeable quantities in silver birch buds only. Some of these compounds have previously been identified in silver birch buds [31], but to the best of our knowledge, kumatakenin, 3'-methoxyapigenin, and cirsimaritin have not previously been found in birch buds.

Species-specific differences in the composition of phenylpropanoids were also observed. Esters of sesquiterpene alcohols and hydroxycinnamic acids were detected only in downy birch buds, although *n*-docosyl *p*-coumarate was characteristic of both species.

### Cytotoxic and antiproliferative activity of bud extracts

The cytotoxicity of *B*. *pendula* and *B*. *pubescens* bud extracts on cancer cells and normal fibroblasts was evaluated by MTT assay. The commonly used anticancer drug cisplatin was used as a reference agent. Cells were treated with extracts for 24, 48 and 72 hours. The concentrations of extracts inducing 50% reduction in cell viability (IC_50_) are shown in Table 2. The IC_50_ values were determined from concentration-response curves presented in Supplementary Information (S2 Fig). All examined extracts induced high concentration- and time-dependent decreases in cell viability. Generally, ether extracts from both birch species exerted lower inhibition of cell viability than SFE or exudates. The most sensitive cell lines compared to fibroblasts were LN-18, MDA-MB-231 and HeLa. The cytotoxic effect of cisplatin was also concentration and time dependent but was stronger than all *Betula* bud extracts. However, the IC_50_ for the reduction of cell viability was generally lower in fibroblasts than in cancer cells.

**Table 2:** Concentrations of extracts (μg/ml ± SEM) required for 50% reduction in cells viability (IC_50_) after 24, 48 and 72 hours of treatment.

**24 hours of treatment**							
**Cell lines**	***B*. *pubescens***			***B*. *pendula***			**cisplatin**
	**SFE**	**exudate**	**ether extract**	**SFE**	**exudate**	**ether extract**	
**LN-18**	**50.23±2.32**	**58.44±2.30**	**81.75±3.55**	**48.32±2.12**	**69.21±3.05**	**83.92±3.62**	**4.51±0.14**
**LN-229**	**62.18±2.89**	**61.58±2.69**	**109.22±4.18**	**55.99±3.43**	**71.15±3.14**	142.50±6.49	33.52±2.80
**MCF-7**	109.9±4.12	88.69±5.22	148.45±6.03	93.89±5.37	102.25±4.67	157.70±6.50	15.34±0.58
**MDA-MB-231**	**54.33±3.15**	**59.53±2.98**	170.00±7.61	**49.07±2.16**	**60.70±2.91**	125.64±7.00	34.51±1.32
**HeLa**	**58.62±2.45**	**63.69±2.26**	**118.00±6.10**	**44.79±2.36**	**75.42±3.12**	143.30±5.21	19.60±0.86
**Ishikawa**	93.56±5.24	88.62±3.12	**123.78±5.28**	85.19±4.21	96.17±4.48	125.82±5.76	36.82±1.26
**AGS**	84.14±3.65	65.42±3.56	141.12±6.32	**69.51±3.32**	88.82±3.44	145.83±8.14	26.41±1.05
**HepG2**	81.31±4.01	76.15±4.05	152.80±6.98	80.12±4.58	93.42±5.12	149.32±7.55	**9.55±0.62**
**DLD-1**	97.57±3.95	75.83±2.66	137.30±6.59	97.36±4.24	90.89±4.56	148.30±6.32	32.25±1.11
**C32**	**63.24±3.88**	79.74±4.07	122.42±7.01	**60.13±2.11**	**79.62±3.43**	**122.41±4.67**	15.90±0.44
**Fibroblasts**	87.99±4.22	70.47±3.02	151.50±8.96	87.64± .45	88.14±4.01	137.33±6.53	14.52±0.87
**48 hours of treatment**							
**LN-18**	**24.01±1.33**	**28.40±1.62**	**55.74±2.30**	**29.84±1.18**	**44.63±1.97**	**60.08±2.89**	**3.20±0.11**
**LN-229**	**36.72±1.89**	53.08±0.67	**66.58±3.01**	**43.55±2.55**	**51.92±2.61**	93.28±4.22	12.53±0.44
**MCF-7**	92.23±4.16	78.92±3.68	106.80±5.19	84.21±4.45	84.69±3.88	109.30±6.04	7.60±0.32
**MDA-MB-231**	**32.39±1.92**	52.73±4.12	**60.46±3.66**	**28.60±1.24**	**49.80±2.38**	**70.63±2.97**	28.10±1.36
**HeLa**	**42.89±2.45**	**43.93±2.17**	109.60±7.23	**41.97±1.95**	**57.89±3.25**	116.10±4.87	11.39± .63
**Ishikawa**	74.61±3.80	78.87±3.27	115.90±5.10	60.00±2.62	86.37±3.76	115.23±6.12	27.10±1.40
**AGS**	**34.68±1.67**	50.58±2.89	135.10±5.84	**41.75±2.30**	**59.23±2.45**	140.20±6.12	21.25±1.12
**HepG2**	49.75±2.25	51.89±2.08	123.40±7.14	55.14± .70	**63.84± .31**	123.00±7.55	**4.25± .12**
**DLD-1**	73.82±3.30	64.44±3.11	109.60±4.23	71.13±4.02	75.25±3.29	120.70±7.90	25.32±0.97
**C32**	51.00±3.14	55.34±2.21	**102.45±4.67**	**43.68±2.90**	**62.22±3.05**	97.68±4.72	6.32±0.24
**Fibroblasts**	52.31±2.91	55.26±2.90	84.52±3.61	57.42±3.50	73.02±4.21	97.18±6.20	5.65±0.30
**72 hours of treatment**							
**LN-18**	**15.94±0.89**	**23.98±1.02**	**21.21±0.90**	**15.84±0.67**	**24.63±1.08**	**24.61±1.32**	2.86±0.12
**LN-229**	**33.73±1.34**	**42.66±1.87**	**42.46±1.64**	**39.26±1.35**	**40.68± .52**	62.26±3.40	5.61±0.18
**MCF-7**	45.76±1.89	64.03±3.06	73.77±3.40	45.00±2.50	62.42±2.69	75.12±4.04	5.21±0.15
**MDA-MB-231**	**23.94±0.98**	49.33±1.67	**43.21±1.82**	**21.84±0.75**	**48.29±1.64**	59.73±2.68	10.25±0.34
**HeLa**	**29.56±1.32**	**36.85±1.44**	**46.57±2.16**	**32.55±2.05**	**35.32±1.59**	58.81±2.68	11.38±0.46
**Ishikawa**	57.64±2.98	69.79±3.68	79.83±3.22	44.29±3.11	57.19±3.04	86.58±4.53	6.48±0.14
**AGS**	**31.02±1.04**	**36.23±1.25**	87.07±3.16	**26.92±0.88**	**37.14±1.08**	80.49±3.76	11.59±0.45
**HepG2**	41.02±2.34	**38.84±1.78**	84.53±3.45	42.71±1.85	**48.86±2.32**	92.33±3.61	3.11±0.11
**DLD-1**	55.99±2.44	58.58±2.18	74.26±3.21	56.63±2.80	57.29±2.46	77.55±3.12	9.73±0.36
**C32**	44.72±2.65	54.26±2.14	88.01±4.57	38.43±2.67	56.58±4.12	65.17±2.77	3.12±0.24
**Fibroblasts**	42.88±2.55	47.83±2.24	59.85±2.69	42.91±2.18	58.62±2.45	61.89±3.26	2.84±0.13

Lower IC_50_ values compared to fibroblasts are in bold.

To determine whether *Betula* bud extracts impair cell proliferation after 24 hour exposure, [^3^H]thymidine incorporation assay was performed. IC_50_ values were determined from the concentration-response curves presented in Supplementary Information (S3 Fig). As can be seen from the data in Table 3, antiproliferative activity of ether extracts was less pronounced than in SFE or exudates. Nevertheless, IC_50_ values of both ether extracts and the *B*. *pubescens* bud exudate for fibroblasts were higher than the values obtained in cancer cells. Of note, these values were lower than IC_50_ for reduction in cell viability, suggesting antiproliferative activity. Further comparison of IC_50_ for [^3^H]thymidine incorporation and IC_50_ for reduction of cell viability revealed that all examined extracts exhibited stronger antiproliferative activity than cytotoxic activity in HeLa and AGS cells. Moreover, almost all tested extracts more efficiently inhibited cell proliferation than induced cell death in MCF-7, Ishikawa, DLD-1 cells (except *B*. *pendula* exudate) and fibroblasts (except *B*. *pendula* ether extract). Cisplatin inhibits cell proliferation stronger than the tested extracts. IC_50_ values for the reduction of [^3^H]thymidine incorporation were a few times lower than the IC_50_ for the reduction of cell viability, but fibroblasts were also one of most susceptible cell types for antiproliferative action of cisplatin.

**Table 3:** Concentrations of extracts (μg/ml ± SEM) required for 50% reduction in [^3^H]thymidine incorporation (IC_50_) after 24 hours of treatment.

Cell lines	*B*. *pubescens*			*B*. *pendula*			cisplatin
	SFE	exudate	ether extract	SFE	exudate	ether extract	
**LN-18**	**59.96±3.22**	**33.34±1.18**	**50.54±2.44**	66.77±5.03	**52.33±2.96**	**73.32±4.61**	2.16± .18
**LN-229**	**70.89±3.18**	56.93±2.34	**98.56±5.32**	77.39±3.40	83.32±5.13	**108.20±6.03**	2.88±0.17
**MCF-7**	78.17±3.37	59.25±2.43	110.00±6.90	68.09±2.45	98.24±4.22	**115.80±4.99**	3.42±0.12
**MDA-MB-231**	**65.36±2.32**	42.84±1.55	124.20±7.72	59.13±2.11	75.68±4.58	**106.70±6.02**	9.12±0.32
**HeLa**	**40.69±1.50**	**20.80±1.20**	**47.82±2.11**	**38.30±1.29**	**49.58±2.80**	**62.50±3.20**	2.64±0.11
**Ishikawa**	77.61±3.21	**35.01±2.90**	**83.99±3.58**	**42.11±2.33**	**97.21±5.91**	**96.46±4.18**	5.56±0.18
**AGS**	**43.23±1.59**	**36.23±1.68**	**91.72±3.90**	**49.97±2.58**	**55.28±3.14**	**127.20±5.22**	4.84±0.14
**HepG2**	77.54±3.02	**33.96±1.69**	**88.51±4.11**	78.07±4.90	87.82±3.86	**107.30±5.85**	3.82±0.21
**DLD-1**	70.85±3.10	**30.01±1.42**	**78.84±3.50**	**45.95±2.77**	86.33±4.52	**103.70±4.62**	4.38±0.19
**C32**	**60.14±2.47**	**40.40±3.21**	**72.55±3.66**	61.17±3.14	82.10±3.51	**98.92±4.06**	**2.21±0.11**
**Fibroblasts**	79.89±3.12	46.52±2.50	116.60±6.11	63.34±2.90	69.25±3.23	142.8±8.45	2.47± 0.13

Lower IC_50_ values compared to fibroblasts are in bold.

## Discussion

In this study, we have demonstrated the potent cytotoxic and differential activities of extracts from buds of *B*. *pendula* and *B*. *pubescens*. The group of compounds that could significantly influence anticancer activity were the triterpenes, which were dominant in all extracts of *B*. *pendula*. Many studies have confirmed that this group of natural components has various potential modes of anticancer action [32]. It has previously been demonstrated that dammarane-type triterpenes from *Betula* spp. possess cytotoxic activity towards Ehrlich carcinoma ascite cells. The action of these compounds was associated with an effect on the microviscosity of tumour-cell membranes [33]. Another important group of compounds with anticancer activity are the flavonoids, especially the methoxylated derivatives of flavones. It was previously shown that *O*-methylation enhanced cytotoxicity on human leukaemia cells [34]. Such compounds were found in most extracts of both birch species but were at higher levels in *B*. *pubescens*. Sesquiterpenoids may also contribute to the cytotoxic activity of birch bud extracts, if not directly, at least indirectly. For example, Legault and Pichette [35] demonstrated that *β*-caryophyllene increased the anticancer activity of other substances.

According to the literature, esters of cinnamic acids with aliphatic alcohols show different biological activities [36,37]. For example, phenylpropenoids of two terpene alcohols (geraniol and farnesol) had an inhibitory effect on nitric oxide production and exhibited antitumour activity [38]. *In vitro* experiments showed that synthetic esters of ferulic and caffeic acids have the ability to inhibit the development of colon, gastric and breast cancer cells [37].

Among the examined cancer cell lines, LN-18, MDA-MB-231 and HeLa were more sensitive to the cytotoxic action of tested extracts than fibroblasts. This indicates that, similar to the anticancer drug used as reference, extracts from *Betula* buds do not show general selectivity towards cancer cells. Nevertheless, further studies are needed for the isolation and identification of pure compounds with anticancer activity.

## Supporting information

S1 Text Analytical procedure. (PDF)

S1 Table Chemical composition of birch bud extracts. S1Table presents the chemical composition of extracts (SFE, exudate, and ether extract of milled buds) and some of the analytical parameters: *I*^T^ values, *m/z* of target peaks, and molecular ion, M^+^ (if was registered).
(PDF)

S1 Fig Chromatograms of SFE extracts of silver birch (upper) and downy birch. (PDF)

S2 Fig Concentration-response curves for the cytotoxic effect of birch bud extracts. The effect of *B*. *pendula* exudate (red closed squares), *B*. *pubescens* exudate (red open squares), *B*. *pendula* SFE (blue closed triangles), *B*. *pubescens* SFE (blue open triangles), *B*. *pendula* ether extract (green closed circles) and *B*. *pubescens* ether extract (green open circles) on cell viability after 24, 48 or 72 hours of treatment. The results are presented as a mean ± SEM of three independent experiments done in triplicates. *P < 0.05.
(PDF)

S3 Fig Concentration-response curves for the antiproliferative effect of birch bud extracts. The effect of *B*. *pendula* exudate (red closed squares), *B*. *pubescens* exudate (red open squares), *B*. *pendula* SFE (blue closed triangles), *B*. *pubescens* SFE (blue open triangles), and *B*. *pendula* ether extract (green closed circles) and *B*. *pubescens* ether extract (green open circles) on [^3^H]thymidine incorporation after 24 hours of treatment. The results are presented as a mean ± SEM of two independent experiments done in triplicates. *P < 0.05.
(PDF)
