# Supplementary material for: Cytotoxicity of white birch bud extracts: Perspectives for therapy of tumours
Source: PLoS One. 2018 Aug 14;13(8):e0201949. doi: 10.1371/journal.pone.0201949 (PMC6091957; doi:10.1371/journal.pone.0201949)
Supplement: S1 Text — (PDF) [file pone.0201949.s001.pdf]

S1 text. Analytical procedure

Obtained extracts of the birch buds were separated and analyzed by a GC–MS method on a HP 7890A gas chromatograph with the 5975C VL MSD Triple-Axis Detector (Agilent Technologies, USA). The apparatus was fitted with an HP-5MS capillary column (30 m × 0.25 mm i. d., 0.25 µm film thickness), with electronic pressure control and split/splitless injector. The later worked at 250°C in the split (1:50) mode. Helium flow rate through the column was 1 mL/min in constant flow mode. Injection of 1 µL of the sample was performed with the aid of autosampler G4513a. The injector (250 ^{o}C) worked in split mode (1:50). The initial column temperature was 50 ^{o}C rising to 310 ^{o}C at 5 ^{o}C/min. The MSD detector acquisition parameters were as follows: the transfer line temperature was 280 ^{o}C, the MS source temperature 230 ^{o}C and the MS quad temperature 150 ^{o}C. The electron impact mass spectra were obtained at 70 eV of ionization energy. Detection was performed in the full scan mode from 41 to 650 a.m.u. After integration, the fraction of separated components in the total ion current (TIC) was calculated.

To identify the components, both mass spectral data and the calculated retention indices were used. Mass spectrometric identification was carried out with an automatic system of GC–MS data processing supplied by NIST 14 library (NIST/EPA/NIH Library of Electron Ionization Mass Spectra) and home-made mass spectra libraries. The latter contains more than 1800 spectra of TMS derivatives prepared from authentic preparations of flavonoids and other phenolics, as well as terpenoids, aliphatic acids, alcohols and carbohydrates.

Hexane solution of C_{10}–C_{40} *n*-alkanes were separated under the above conditions values. The retention indices (I^{T}) of the registered components were calculated from the results of the separation of this solution and silanized bud extracts using the following equation:

$I^{T} = 100(t_x – t_n)/(t_{n+1} – t_n) + 100n$

where t_x, t_n, and t_{n+1} are the retention times of compounds x and n-alkanes with the number of carbon atoms in the molecule n and $n + 1$, respectively ($t_n \leq t_x \leq t_{n+1}$) (Van den Dool and Kratz, 1963). The calculated I^T values were compared with NIST collection (NIST 2013) as well as with the authors' previously published data (Isidorov and Szczepaniak, 2009; Isidorov et al. 2014; Isidorov, 2015; Isidorov et al., 2016). The identification was considered reliable if the results of computer search at the mass spectra library were confirmed by the experimental I^T values, i.e. if their deviation from the averaged literature values did not exceed ±10 u.i. (inter-laboratorial deviation for low polar stationary phases). The composition of extracts of birch buds was very complex: on the chromatograms of six extracts, 150 substances belonging to different groups of organic compounds were registered. Chemical composition of extracts was species specific as can be seen from chromatograms of SFE extracts of downy birch (*B. pubescens*) and silver birch (*B. pendula*) buds (S1Fig).

Van den Dool H, Kratz PD. A generalization of the retention index system including linear temperature programmed gas—liquid partition chromatography. J. Chromatogr. 1963; 11: 436–471.

NIST Chemistry WebBook, 2013. National Institute of Standards and Technology, Gaithersburg, MD 20899, http://webbook.nist.gov.chemistry.

Isidorov VA, Szczepaniak L. Gas chromatographic retention indices of biologically and environmentally important organic compounds on capillary columns with low-polar stationary phases. J Chromatogr A. 2009; 1216: 8998–9007.

Isidorov V, Szczepaniak L, Bakier S. Rapid GC/MS determination of botanical precursors of Eurasian propolis. Food Chem. 2014; 142:101–110.

Isidorov VA. Identification of Biologically and Environmentally Significant Organic Compounds. Mass Spectra and Retention Indices of Trimethylsilyl Derivatives. PWN, Warsaw, 2015, 430 pp.

IsidorovVA, Bakier S, PirożnikowE, Zambrzycka M, Swiecicka I. Selective behaviour of honeybees in acquiring European propolis plant precursors. J. Chem. Ecol. 2016; 42: 475-485.
