# Supplementary material for: Cytotoxicity of white birch bud extracts: Perspectives for therapy of tumours
Source: PLoS One. 2018 Aug 14;13(8):e0201949. doi: 10.1371/journal.pone.0201949 (PMC6091957; doi:10.1371/journal.pone.0201949)
Supplement: S1 Table — S1Table presents the chemical composition of extracts (SFE, exudate, and ether extract of milled buds) and some of the analytical parameters: IT values, m/z of target peaks, and molecular ion, M+ (if was registered). (PDF) [file pone.0201949.s002.pdf]

S1 Table. Chemical composition (% of TIC) and some analytical parameters of birch bud extracts

**Compound**	**Analytical parameters**			***B. pubescens***			***B. pendula***		
	$\boldsymbol{I^T}$	**Target ions (*m/z*)**	$\mathbf{M^+}$	**SFE**	**Exud**	**Extr**	**SFE**	**Exud**	**Extr**
Lactic acid, di-TMS	1069	147,73,117,190,191	-	-*	-	0.02	-	-	-
Benzyl alcohol, mono-TMS	1155	165,91,135,73,65	180	0.05	-	-	-	-	-
Malonic acid, di-TMS	1217	147,73,233,75,148	248	-	-	-	-	0.05	trace[2*]
2-Phenyl ethanol, mono-TMS	1227	179,73,103,105,75	-	0.05	-	-	-	-	-
H_3PO_4, tri-TMS	1290	299,73,300,211,314	314	-	-	0.01	-	trace	trace
Glycerol, tri-TMS	1293	73,147,205,133,117	-	-	-	0.01	-	0.02	trace
Succinic acid, di-TMS	1324	147,73,247,172,129	262	-	-	0.01	-	-	-
α-Copaene	1378	119,161,105,93,91	204	-	-	-	0.07	0.09	0.03
β-Caryophyllene	1418	93,133,91,41,69	204	0.36	0.11	0.13	0.09	0.17	0.11
Birkenal	1440	93,105,69,41,79	206	0.43	1.01	0.42	-	-	-
α-Humulene	1454	93,80,121,41,147	204	0.12	-	0.03	-	0.05	0.06
Germacrene D	1480	161,105,91,119,93	204	-	-	-	0.01	0.20	0.19
γ-Cadinene	1514	161,105,91,204,79	204	-	-	-	0.01	trace	-

δ-Cadinene	1522	161,134,119,105,204	204	-	-	-	0.09	0.06	-
Anisic acid, mono-TMS	1522	209,135,165,224,92	224	-	-	0.03	-	-	-
Birkenol, mono-TMS	1561	73,75,191,143,119	280	0.14	-	-	-	-	-
Sesquiterpenol $C_{15}H_{26}O$, mono-TMS	1566	73, 93,95,75,187	294	0.11	0.15	0.07	-	-	-
Caryophylla-4(12),8(13)-dien-5-one	1574	134,69,41,91,79	218	-	-	0.01	-	-	-
Caryophyllene oxide	1577	79,43,41,93,95	220	0.90	0.48	0.32	0.28	0.19	0.05
4-Hydroxyphenylethanol, mono-TMS	1582	179,73,282,267,193	182	-	-	-	-	0.12	0.24
Humulene epoxide II	1603	43,67,41,138,109	220	0.16	0.05	0.03	0.05	trace	-
1-*epi*-Cubebol, mono-TMS	1617	279,161,73,75,143	294	-	-	-	trace	trace	-
epi-Cubebol, mono-TMS	1627	279,75,161,204,143	294	-	-	-	0.05	-	-
Sesquiterpenol $C_{15}H_{24}O$, mono-TMS	1629	73,75,131,156,41	292	1.18	-	-	-	-	-
Caryophylladien-5α-ol, mono-TMS	1637	73,156,75,131,169	292	0.18	0.78	0.59	-	-	-
Sesquiterpenol $C_{15}H_{24}O$, mono-TMS	1641	73,41,131,156,75	292	-	0.13	0.11	-	-	-
Betulenal	1645	91,69,41,79,105	218	0.18	-	0.06	-	-	-
Caryophylladien-5β-ol, mono-TMS	1658	73, 75,131, 156,169	292	0.11	-	0.04	-	-	-
Sesquiterpenol $C_{15}H_{24}O$, mono-TMS	1664	41,79,91,69,55	292	-	-	0.04	-	-	-

Sesquiterpenoid	1677	109,135,123,93,91	-	-	0.07	0.05	-	-	-
6-Hydroxy-β-caryophyllene, mono-TMS	1682	73,156,131,169,189	292	12.54	5.93	5.80	1.48	2.85	2.38
Sesquiterpenol $C_{15}H_{24}O$, mono-TMS	1691	73,208,75,41,91	292	0.28	0.19	0.18	-	-	-
14-Hydroxy-β-caryophyllene, mono-TMS	1705	73,144,209,75,181	292	3.49	1.45	1.57	0.45	0.77	0.62
Caryophylla-3,8(13)-dien-5α-ol acetate	1714	131,43,105,187,91	262	0.31	0.14	0.09	0.25	-	-
14-Hydroxy-β-isocaryophyllene, mono-TMS	1723	73,155,131,91,181	292	2.12	0.78	0.75	-	0.39	0.71
Caryophylla-4(12),8(13)-dien-5β-ol acetate	1738	131,91,43,105,93	262	0.11	-	0.03	-	-	-
α-Cadinol, mono-TMS	1746	143,209,161,204,73	294	-	-	-	-	0.29	0.09
6-Hydroxy-β-caryophyllene acetate	1754	133,43,69,91,131	262	2.28	1.07	0.79	0.31	trace	-
3-Hydroxy-β-caryophyllene acetate	1761	43,131,93,146,187	262	0.76	0.30	0.29	0.09	-	-
14-Hydroxy-α-humulene acetate	1772	43,131,80,187,41	-	1.61	0.74	0.64	-	-	-
Sesquiterpenoid?[3*], TMS	1781	73,132,121,177,93	-	-	0.07	0.06	-	-	-
14-Hydroxy-β-caryophyllene acetate	1790	91,43,69,105,93	262	10.72	5.47	3.97	1.60	trace	-
Sesquiterpenoid $C_{15}H_{24}O_2$-mono-TMS	1799	73,132,121,75,177	308	2.82	1.27	1.08	-	-	-
Sesquiterpenoid $C_{15}H_{24}O_2$-mono-TMS	1802	109,73,183,132,43	308	-	0.83	0.48	-	-	-
Sesquiterpenoid $C_{15}H_{24}O_2$-mono-TMS	1814	73,132,121,119,41	308	1.88	1.29	0.76	-	-	-

Sesquiterpenoid?	1819	73,109,183,143,240	-	-	0.10	0.08	-	-	-
NN[4*]	1852	251,73,271.75	-	0.22	0.13	0.13	-	-	-
Sesquiterpenoid $C_{15}H_{24}O_2$-mono-TMS	1858	73,103,129,93,41	308	1.06	0.79	0.52	-	-	-
Caryophylla-4(14),8(15)-diene, 5,6-dihydroxy-, di-TMS, isomer 1	1874	73,208,143,147,221	380	0.41	0.29	0.23	-	-	-
Sesquiterpenoid $C_{15}H_{24}O_2$-di-TMS	1884	73,169,147,191,143	380	0.57	036	0.39	-	-	-
Sesquiterpenoid $C_{15}H_{24}O_2$-di-TMS	1891	209,75,117,143,185	380	0.25	0.12	0.12	-	-	-
Sesquiterpenoid $C_{15}H_{24}O_2$-di-TMS	1897	73,156,169,143,275	380	0.71	0.77	045	-	-	-
Caryophylla-4(14),8(15)-diene, 5,6-dihydroxy-, di-TMS, isomer 2	1902	73,208,147,143,221	380	3.28	4.61	2.70	-	-	-
Sesquiterpenoid $C_{15}H_{24}O_2$-di-TMS	1916	73,143,91,181,275	380	0.35	0.24	0.12	-	-	-
Sesquiterpenoid $C_{15}H_{24}O_2$-di-TMS	1919	73,43,277,93,131	380	-	-	0.37	-	-	-
Sesquiterpenoid $C_{15}H_{24}O_2$-di-TMS	1922	73,147,269,204,191	380	trace	-	0.36	-	-	-
14-Hydroxy-4,5-epoxy-β-caryophyllene acetate	1924	43,93,79,91,108	-	1.30	1.01	0.77	-	-	-
Sesquiterpenoid $C_{15}H_{24}O_2$-di-TMS	1934	73,143,147,93,181	380	-	-	0.10	-	-	-
p-Coumaric acid, mono-TMS	1947	293,219,308,73,249	308	0.38	048	0.42	-	0.05	trace

NN	1955	73,43,118,205,75	-	0.41	0.45	0.66	-	-	-
NN	1979	73,43,117,41,75	-	0.71	0.42	0.39	-	-	-
Gallic acid	1985	281,458,73,443,179	458	-	-		-	0.21	0.02
5,6,8-Trihydroxycaryolan	1988	251,204,73,359,147	470	2,58	3.90	2.85	-	-	-
NN	1998	73,147,269,470	-	-	0.15	0.14	-	-	-
NN	2014	73,75,143,443	-	-	0.08	0.12	-	-	-
Hexadecanoic acid, mono-TMS	2052	313,117,73,75,129	328	0.36	-	0.15	0.82	0.77	0.25
Sesquiterpenoid $C_{15}H_{24}O_2$-di-TMS	2059	251,269,73,380	380	-	-	0.05	-	-	-
Sesquiterpenoid $C_{15}H_{24}O_2$-di-TMS	2095	73,143,147,380	380	-	-	0.07	-	-	-
n-Heneicosane	2100	57,71,43,85,41	296	trace	0.14	0.13	-	-	-
NN	2128	73,191,147,75...468	-	-	0.28	0.22	-	-	-
Linoleic acid, mono-TMS	2215	73,75,67,81,129	352	0.89	trace	0.02	2.66	2.88	1.67
Oleic acids, mono-TMS	2221	73,75,117,129,55	354	0.57	-	0.07	1.65	1.55	1.37
α-Linolenic acids, mono-TMS	2223	75,73,79,67,95	350	0.72	-	0.07	2.00	1.05	2.07
NN	2239	143,73,147...468	-	-	0.14	0.12	-	-	-
Octadecanoic acid, mono-TMS	2249	341,117,73,75,132	356	0.17	-	0.02	0.22	trace	trace

n-Hexyl *p*-coumarate, mono-TMS	2292	236,219,320,73,192	320	0.03	-	0.06	-	-	-
n-Tricosane	2300	57,85,71,43,41	324	1.08	0.95	0.47	0.45	0.04	0.12
1-Eicosanol, mono-TMS	2360	355,75,103,43,57	-	-	-	-	-	0.07	0.06
Eicosanoic acid, mono-TMS	2448	369,117,73,132,129	384	0.40	0.07	0.17	0.09	0.23	0.10
n-Pentacosane	2500	57,85,71,43,41	352	0.97	0.75	0.37	5.05	0.46	1.41
1-Docosanol, mono-TMS	2556	383,384,75,43,103	-	0.46	0.28	0.33	1.2	0.55	0.27
Docosanoic acid, mono-TMS	2645	397,117,73,129,132	412	0.93	0.19	0.40	0.21	0.13	0.10
n-Heptacosane	2700	57,71,85,43,41	380	1.57	1.24	0.49	4.22	1.14	0.97
1-Tetracosanol, mono-TMS	2754	411,75,57,43,103	-	0.29	-	0.20	0.30	0.54	0.16
Unidentified non-completely derivatized flavonoid	2773	179,192,358,177,73	358?	3.877	0.21	0.23	-	-	-
4',7-Dimethylnaringenin TMS	2789	367,238,368,73,134	372	2.50	2.50	2.12	0.13	0.13	0.13
n-Octacosane	2800	57,85,71,43,41	-	-	-	-	0.08	-	-
Isosakuranetin, di-TMS	2815	415,416,73,296,429	430	0.19	0.56	0.92	-	-	-
Chalcone?	2830	222,73,311,460,445	460	-	0.15	0.16	-	-	-
Hexacosanal	2836	82,57,43,96,55	-	-	-	-	0.09	trace	-
Tetracosanoic acid, mono-TMS	2844	425,117,73,132,145	440	0.49	0.21	0.38	0.18	0.23	0.19

Sakuranetin, di-TMS	2880	415,238,416,73,238	430	6.14	14.6	12.5	0.13	0.33	0.63
Naringenin, tri-TMS	2898	473,296,73,474,179	488	-	0.76	1.14	-	-	-
n-Nonacosane	2900	57,85,71,43,41	-	0.14	-	-	0.22	0.02	0.09
Unidentified chalcone or flavonoid	2915	280,73,311,281,503	518	0.42	1.10	1.04	-	-	-
4',7-Dimethylapigenine (non-Sil)	2925	298,255,299,269,297	298	1.16	-	0.19	-	-	-
Catechine, penta-TMS	2936	368,355,73,369,356	650	-	-	trace	0.29	0.35	0.28
1-Hexacosanol, mono-TMS	2951	439,440,75,43,103	-	013	-		0.10	0.09	trace
Flavonoid?	2962	371,372,73,328,386	386?	1.47	-	-	-	-	-
Acacetin, mono-TMS	2990	356,341,356,75,73	356	0.59	0.40	0.48	-	0.04	0.15
Rhamnocitrin, di-TMS	3011	429,386,430,73,135	444	1.27	0.33	-	-	0.34	0.42
Kumatakenin, di-TMS	3030	443,444,73,445,371	458	1.96	4.15	3.25	-	-	-
Hexacosanoic acid, mono-TMS	3042	453,117,73,129,132	468	0.58	0.26	-	0.50	0.69	0.35
3'-Methoxyapigenin, tri-TMS	3049	501,502,73,503,458	516	0.54	4.69	6.45	-	0.34	0.46
Ermanin, di-TMS	3062	443,444,73,445,385	458	0.57	4.60	7.94	-	0.45	0.29
Acacetin, di-TMS	3066	413,414,73,370,415	428	-	4.41	-	-	trace	-
Caryophylladien-5α-ol *p*-coumarate, mono-TMS	3089	219,73,307,290,220	438	0.12	0.10	0.47	-	-	-

Rhamnocitrin , tri-TMS	3096	501,502,503,73,429	516	0.36	3.19	4.78	-	0.10	0.12
Kaempherol, tetra-TMS	3114	559,560,73,561,487	574	-	1.56	2.68	-	-	-
Quercetin, dimethyl ether, tri-TMS	3118	531,532,73,501,386	-	0.40	0.62	-	0.30	1.02	0.78
Cirsimaritin (6-methoxyapigenin), di-TMS	3135	443,444,73,445,75	458	-	1.44	1.75	0.46	0.99	0.86
6-Hydroxycaryophyllene *p*-coumarate, mono-TMS	3136	219,220,73,236,423	438	1.70	2.29	3.54	-	-	-
Triterpenoid TMS?	3140	416,73,415,397	-	-	-	-	0.52	0.12	-
1-Octacosanol, mono-TMS	3148	467,75,57,43,103	-	0.27	-	-	0.13	0.35	0.28
Apigenin, tri-TMS	3162	471,472,473,73,228	486		0.68	1.08	-	-	-
14-Hydroxycaryophyllene *p*-coumarate, mono-TMS	3164	219,236,73,205,159	438	1.01	0.94	2.25	-	-	-
14-Hydroxyisocaryophyllene *p*-coumarate, mono-TMS	3182	219,119,134,236,73	438	0.67	0.35	0.88	-	-	-
Dimethylquercetin, tri-TMS, isomer 1	3207	531,532,533,73,501	-	-	0.34	0.54	-	-	-
Triterpenoid	3225	189,129,145,117,397	-	1.85	0.98	1.43	-	-	-
4-Hydroxycynnamyl *p*-coumarate, TMS	3241	117,356,73,307,115	440	-	1.66	-	-	-	-
Octacosanoic acid, mono-TMS	3241	481,117,73,145,132	496	0.58	-	-	0.13	0.23	0.14
Dimethylquercetin, tri-TMS, isomer 2	3246	531,532,533,73,501	546	-	1.66	4.59	-	-	-

Triterpenoid, TMS	3252	69,73,367,219,...512	512	-	-	-	0.29	0.23	0.40
Lup-20(29)-en-28-al?	3269	203,41,69,55,81	424	-	-	-	16.0	28.35	35.29
Dammaradien-3-one	3306	109,69,205,95,93	424	-	-	-	5.01	5.00	4.08
6-Hydroxycaryophyllene ferulate, TMS	3309	249,219,73,91,236	468	0.16	0.18	0.16	-	-	-
14-Hydroxycaryophyllene ferulate, TMS	3332	249, 219,73,250,220	468	0.23	-	0.16	-	-	-
Triterpenoid	3343	189,109,101,190,95	498	-	-	-	0.57	1.23	0.97
14-Hydroxy α-humulene ferulate, TMS	3346	249,266, 250,219,119	468	0.06	-	-	-	-	-
β-Sitosterol, mono-TMS	3348	129,357,396,381,73	486	0.25	-	0.21	0.19	0.98	1.05
14-Hydroxycaryophyllene caffeate, di-TMS	3362	219,73,236,307,91	526	0.13	0.20	0.22	-	-	-
Coniferyl *p*-coumarate, mono-TMS	3400	219,73,220,235,204	470	-	0.15	0.35	-	-	-
Triterpenoid, TMS	3420	199,69,73...505	-	-	-	-	1.25	3.65	3.54
Triterpenoid, TMS	3431	189,129,145,117,73	-	0.42	-	0.23	-	-	-
Triterpenoid, TMS	3441	109,121,123,177,73	-	-	-	-	1.76	7.17	6.47
Triterpenoid	3448	123,121,177,189,109	424	-	-	-	0.70	2.87	2.66
Triterpenoid, TMS	3504	199,69,73,431	-	-	-	-	1.66	2.45	2.34
Dipterocarpol, mono-TMS	3510	199,69,73,143,431	-	0.12	-	0.07	7.06	8.90	8.33

Triterpenoid	3541	189,73,129	-	0.14	-	0.08	2.12	1.10	1.11
Betulinic acid, di-TMS	3590	73,189,190,203,129	600	-	-	0.06	-	0.02	1.74
Triterpenoid, TMS	3598	189,199,73	-	-	-	-	0.65	1.17	1.47
Triterpenoid, TMS	3614	131,199,73,215	-	-	-	-	1.92	1.32	1.78
Triterpenoid, TMS	3625	199,69,73,175	-	-	-	-	3.93	2.37	0.35
Triterpenoid, TMS	3688	203,187,43,73,482	-	-	-	-	1.10	2.18	0.31
Triterpenoid acetate?	3709	108,135,43,69,422	422	-	-	-	1.57	0.15	0.34
Triterpenoid, TMS	3740	199,69,422,489,73	-	-	-	-	18.1	7.09	6.17
Triterpenoid, TMS	3766	199,69,43, 73,75	-	-	-	-	1.52	1.08	-
Triterpenoid, TMS	3855	131,73,215	-	-	-	-	1.10	0.43	-
Triterpenoid, TMS	3869	131,215,73	-	-	-	-	1.38	0.54	-
Docosyl *p*-coumarate, mono-TMS	3934	219,236,179,192,237	544	0.7	0.50	0.47	0.87	0.16	0.83
Docosyl palmitate	3972	257,57,43,69,83	564	0.20	-	-	-	-	-

[*]"–" not registered; [2*] "trace" - below 0.01% of TIC; [3*]"?" - tentative; [4*] NN - not identified.
