# Supplementary figures and images for: Cytotoxicity of white birch bud extracts: Perspectives for therapy of tumours
Source: PLoS One. 2018 Aug 14;13(8):e0201949. doi: 10.1371/journal.pone.0201949 (PMC6091957; doi:10.1371/journal.pone.0201949)

Abundance

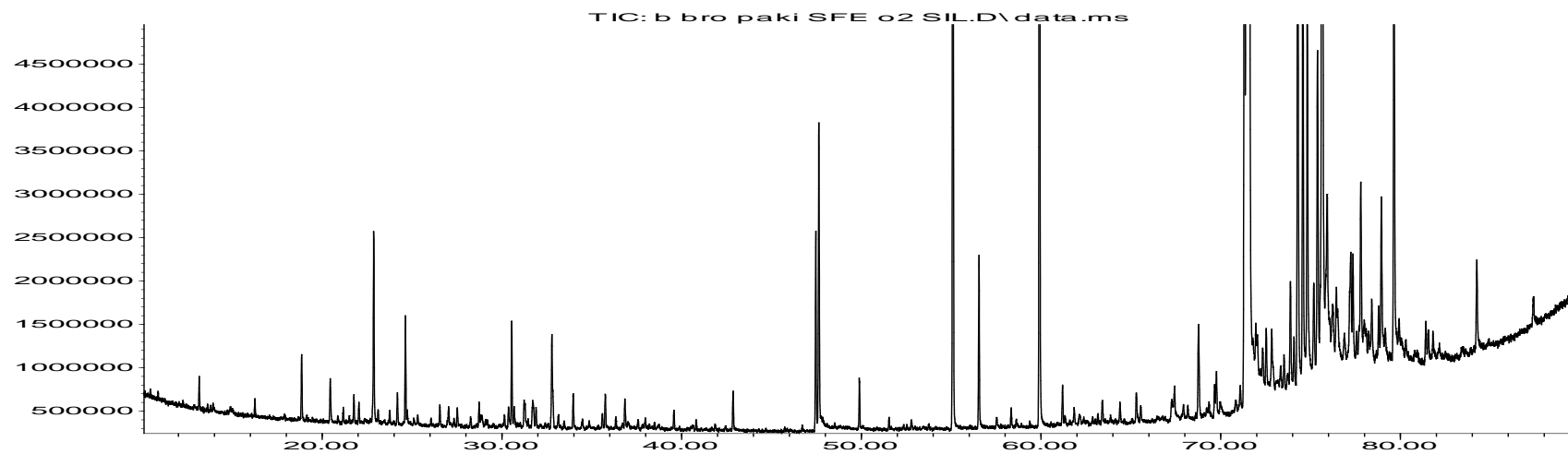

Abundance

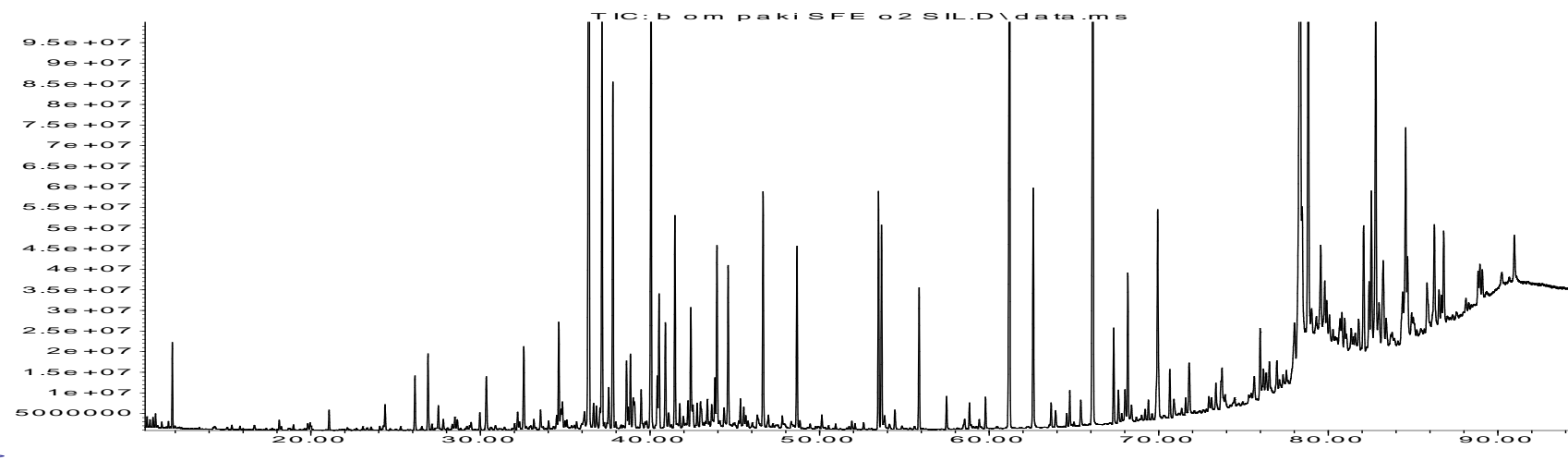

**S1 Fig.** Chromatograms of SFE extracts of silver birch (upper) and downy birch.

Supplement: S1 Fig — (PDF) [file pone.0201949.s003.pdf]
