# Supplementary material for: Cytotoxicity of white birch bud extracts: Perspectives for therapy of tumours
Source: PLoS One. 2018 Aug 14;13(8):e0201949. doi: 10.1371/journal.pone.0201949 (PMC6091957; doi:10.1371/journal.pone.0201949)
Supplement: S3 Fig — The effect of B. pendula exudate (red closed squares), B. pubescens exudate (red open squares), B. pendula SFE (blue closed triangles), B. pubescens SFE (blue open triangles), and B. pendula ether extract (green closed circles) and B. pubescens ether extract (green open circles) on [3H]thymidine incorporation after 24 hours of treatment. The results are presented as a mean ± SEM of two independent experiments done in triplicates. *P < 0.05. (PDF) [file pone.0201949.s005.pdf]

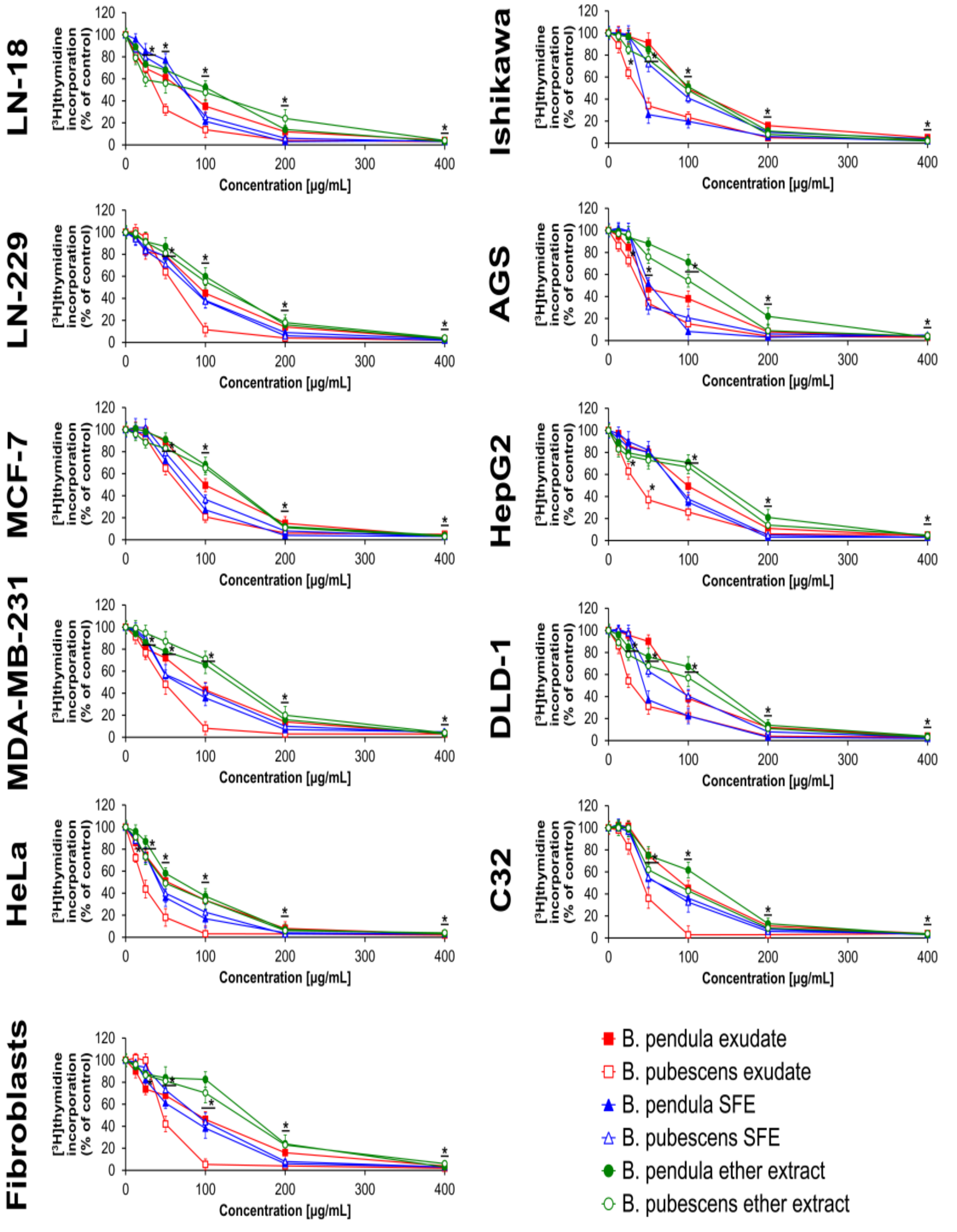

S3 Fig. Concentration-response curves for the antiproliferative effect of birch bud extracts. The effect of *B. pendula* exudate (red closed squares), *B. pubescens* exudate (red open squares), *B. pendula* SFE (blue closed triangles), *B. pubescens* SFE (blue open triangles), and *B. pendula* ether extract (green closed circles) and *B. pubescens* ether extract (green open circles) on [^{3}H]thymidine incorporation after 24 hours of treatment. The results are presented as a mean ± SEM of two independent experiments done in triplicates. *P < 0.05.
